# Evaluating Silicon Application Strategies for Sweet Basil Growth and Postharvest Chilling Injury Alleviation

**DOI:** 10.3390/plants15182755

**Published:** 2026-09-09

**Authors:** Vivian Ly, Youbin Zheng

**Affiliations:** School of Environmental Sciences (SES), Ontario Agricultural College, University of Guelph, Guelph, ON N16 2W1, Canada; vly03@uoguelph.ca

**Keywords:** basil, chilling injury, postharvest quality, silicon

## Abstract

The cold sensitivity of sweet basil often results in unmarketable chilling injury (CI) symptoms and a shorter shelf life under the low temperatures typically used to maintain most fresh produce during transportation and storage. Silicon (Si) treatments have been observed to alleviate these symptoms and provide other benefits during cultivation. This study tested different Si application strategies on basil plants to determine their efficacy at reducing CI symptoms and improving growth, and whether efficacy is influenced by dose–response or Si content in leaf tissues. Across four trials, preharvest treatment solutions containing potassium silicate or monosilicic acid were applied to potted basil at Si rates of 0, 50, 100, 150, and 200 mg/L through either regular rootzone fertigation or weekly foliar spray. There were no significant improvements in growth found at harvest. Although the median shelf life during cold storage at 4 °C ranged between 17 and 24 days for all groups, exceeding known estimates for untreated basil, there were no significant improvements compared to the controls. Drought stress could have been a contributing factor that hardened controls against chilling even without Si applications. Further investigations are necessary to determine the sole effects of Si applications on basil.

## 1. Introduction

Sweet basil (*Ocimum basilicum* L.) is widely cultivated for its high economic value and demand as a popular culinary herb [1]. Being a cold-sensitive crop, basil presents challenges to cold chain logistics that limit its distribution potential. The cold chain is a temperature-controlled supply chain that conserves the food safety and quality of perishable food using refrigeration operations that keep goods at appropriate temperatures during transportation and storage from their point of origin to the consumer. Due to commodities having different optimal temperature ranges, using the more economical and efficient method of shipping and storing in mixed loads may not always be possible, or intermediary temperatures may be used which are sub-optimal for some of the goods. The quality of most herbs and green vegetables is best preserved using temperatures around 0–2 °C; however, temperatures ≤ 10 °C can lead to chilling injury (CI) for basil depending on exposure duration [2,3,4]. Discolouration and necrosis (lesions and dark spots) are common CI symptoms for basil that reduce its commercial quality and shelf life, leading to fewer sales and more food waste [2,4]. The shelf life of basil can range from 1 to 3 days at common refrigeration temperatures of ≤5 °C, preventing wider distribution that requires longer transportation times [4]. This also prevents the use of fresh basil in refrigerated products such as salad mixes, which stay on refrigerated shelves in-store. To our knowledge, no method to improve the cold tolerance of basil has been widely adopted by industry, particularly for the alleviation of CI symptoms during postharvest.

Beneficial effects of silicon (Si) applications, such as reductions in CI symptoms, have been reported during cultivation and postharvest; however, most studies were performed on growing plants during cultivation, and postharvest studies have largely been limited to fruits [5,6]. This leaves knowledge gaps regarding the effects of Si applications on cold-sensitive vegetables during postharvest, such as on basil leaves. The physiological role of Si differs between the cultivation and postharvest stages, necessitating separate investigations to fully understand its contributions during each stage. During cultivation, plants function as integrated systems in which Si supports the coordinated interactions among plant organs vital for growth and adaptation to stressors, such as by enhancing structural development and maintaining homeostasis [7]. During postharvest, the harvested tissues are no longer reliant on coordinated interactions among plant organs, and the focus is instead on preserving cellular integrity and compartmentalization to limit leakage of water and cellular components [8]. Our previous research demonstrated that Si application on basil during cultivation resulted in noticeably reduced development of leaf necrosis (dark spots and lesions) compared to the untreated basil after 2 weeks of postharvest cold storage at 4 °C [9]. The study used Si solutions made from potassium silicate at a Si application rate of 189 mg/L applied by rootzone fertigation or weekly foliar sprays over a 5-week period, with both application methods yielding similar results. An earlier study, although not on postharvest, found that adding potassium silicate at a Si application rate of 75 mg/L in nutrient solutions could reduce damage and death of basil during a cold shock event [10].

From the available information, there are still many variables that require investigation before developing an efficient strategy for Si treatments on basil. It is important to determine whether there is a dose–response relationship that can be used to determine an optimal application rate for the best possible alleviation of CI symptoms or a minimal rate to minimize product use while still observing significantly reduced symptoms. The dose–response can also be dependent upon the chemical form of Si used [11]. Although most plants, particularly dicots, have low capacity for Si uptake, some studies have shown that even non-accumulators or micro-accumulators of Si (<1000 mg/kg) like basil may still see varying benefits from supplemental Si applications, such as increased height, biomass, and chlorophyll contents [10,12,13]. To our knowledge, no studies have determined methods for maximizing Si uptake in basil and whether it influences the efficacy of treatments.

To fill these knowledge gaps, the objective of this study was to evaluate the response of container-grown basil to different Si application strategies. The strategies consisted of a range of Si application rates supplied using different Si-containing fertilizers and applied either through rootzone fertigation or weekly foliar sprays. With the expectation that each strategy will reduce postharvest CI symptoms and potentially improve plant growth during cultivation, we aimed to determine whether the efficacy of each strategy exhibited a dose-dependent response to Si application rate or was related to the Si content in leaf tissues. As basil is not known to accumulate Si, we hypothesized that treatment efficacy may not increase proportionally with increasing Si application rate or leaf Si content.

## 2. Materials and Methods

### 2.1. Plant Materials and Growing Conditions

Sweet basil (*Ocimum basilicum* L. ‘Genovese’) seeds (High Mowing Organic Seeds, Wolcott, VT, USA) were sown in 6″-azalea pots (~20 seeds/pot) containing peat-based soilless growing media (BM6 All-Purpose HP; Berger, Saint-Modeste, QC, Canada) on 6 February 2025. Pots were placed on bench tops in a greenhouse (Edmund C. Bovey Building, University of Guelph, Guelph, ON, Canada). The greenhouse was under natural light supplemented as necessary with high-pressure sodium lights, with a total average daily light integral (DLI) of 7.4 mol/m^2^·d and a 16 h/day photoperiod, temperatures of 23/20 °C (day/night), and relative humidity (RH) of 45/48% (day/night). Irrigation with tap water until leachate appeared occurred once every 1–2 days until treatments began. Treatments started at ~3 weeks after seeding when plants developed 2–4 pairs of true leaves and pots were thinned to 10 plants/pot. All plants were then irrigated until leachate appeared with nutrient solution instead of tap water, which was provided as often as necessary for growing media to remain moist (typically once every 1–2 days). The nutrient solution was made with 1 g of fertilizer (Plant Prod 20-8-20 All Purpose High Nitrate Water Soluble Fertilizer; Master Plant Prod Inc., Brampton, ON, Canada) per litre of tap water to achieve concentrations of 200 mg N/L, 80 mg P_2_O_5_/L, and 200 mg K_2_O/L. The pots were randomly arranged on a section of a bench for each trial and rotated weekly until harvest on 4 April 2025 (~8 weeks after seeding). Pots were sprayed with tap water using a hose the morning of harvest to rinse residual materials from plants before harvesting.

### 2.2. Silicon Treatments

Four trials were conducted to evaluate plant growth at harvest and postharvest quality during cold storage in response to a range of Si application rates applied using different strategies (Table 1). The Si application rates were 0, 50, 100, 150, and 200 mg elemental Si/L (or 0, 1.78, 3.56, 5.34, and 7.12 mmol Si/L, respectively). The strategies consisted of supplying Si as either potassium silicate (KS; K_2_SiO_3_) or monosilicic acid (MSA; H_4_SiO_4_) to the nutrient solution for rootzone fertigation or to tap water for a weekly foliar spray. Rootzone fertigation until leachate appeared was done by hand to each pot as often as necessary to keep growing media moist, typically once every 1–2 days. Foliar sprays were applied to foliage using mister spray bottles until runoff. To prevent contamination from other treatment solutions during foliar sprays, pots from the same treatment group were taken to a separate room when applying. Every trial had 13 replicate pots for each application rate of 50, 100, 150, and 200 mg/L and 8 replicate control pots (0 mg/L), for a total of 60 pots/trial.

The KS was sourced from a commercial liquid Si fertilizer containing 2% soluble Si (Pro-Tekt 0-0-3; SUPERthrive, Los Angeles, CA, USA). The amount of potassium added to treatment solutions by this KS fertilizer in trials 1 and 2 is approximately 43, 87, 130, and 174 mg K/L for the 50, 100, 150, and 200 mg/L treatments, respectively. The MSA was sourced from another commercial liquid Si fertilizer containing 15–20% Si (ZumSil; Black Swallow Soils, Brantford, ON, Canada). This Si fertilizer provides no other nutrients. The final pH of the treatment solutions was adjusted to ~5.5 using 6 M hydrochloric acid, which is the optimal pH to prevent the precipitation of Si and some of the other nutrient elements out of the solution [9]. The amount of chloride added to the treatment solutions ranged from 0.15 to 0.40 mg/L, an amount unlikely to cause adverse effects on plants.

Samples of the treatment solutions were sent to a commercial laboratory (SGS Canada Inc., Guelph, ON, Canada) to determine the measured Si concentration. The nutrient solution and tap water both had 5 mg/L of Si prior to any additions. The treatment solutions had an average Si concentration of 35 or 96 mg/L per mL of KS or MSA, respectively. These rates were used to formulate the treatment solutions for each Si application rate (Table 2).

### 2.3. Analysis of Silicon Content in Basil Leaves

The Si in basil leaf tissues was solubilized using an oven-induced tissue-digestion method and measured using molybdenum blue colourimetry according to the protocol described in Langenfeld & Bugbee [14], with minor deviation when preparing the calibration curve to detect much lower Si concentrations. Three replicate pots/application rates were used from each trial. A replicate consisted of the leaves with their petioles collected from all plants in a pot at harvest.

For the tissue digests, leaves were oven-dried at 80 °C and then finely ground. Dried, ground leaf tissue (100 mg) from each replicate was transferred to its own centrifuge tube, with 5 drops of octyl-alcohol added to reduce foaming and 2 mL of hydrogen peroxide to begin the first digestion. The centrifuge tubes were then capped and placed upright in an oven at 95 °C for 30 min. For the second and final digestion, 4 mL of 12.5 M sodium hydroxide was added to the first digestion and vortexed. The centrifuge tubes were recapped and placed upright in the oven for an additional 4 h. To form monosilicic acid, 1 mL of 5 mM ammonium fluoride was added to the digests, and then 9 mL of 6 M hydrochloric acid was added to neutralize it. Deionized (DI) water was added up to a final volume of 50 mL.

Silicon content was determined using molybdenum blue colourimetry. Aliquots of the final digests were taken to make 10 mL 1:25 dilutions in glass test tubes with DI water (0.4 mL of a digest and 9.6 mL of DI water). Six drops of 6 M hydrochloric acid, 12 drops of 81 mM ammonium molybdate, 8 drops of 1.1 M oxalic acid, and 100 mg of 90% (*w*/*w*) sucrose and 10% (*w*/*w*) isoascorbic acid were added to the dilutions, capping and inverting the test tubes between steps to mix. Cuvettes were filled with these solutions. A calibration curve of 0–1 mg/L of silica was prepared and analyzed using a spectrophotometer (SPECTROstar Nano; BMG LABTECH, Ortenberg, Hessen, Germany). The resulting trendline equation (y = 0.5856x + 0.0278; R^2^ = 0.954) from the calibration curve was used to calculate the concentration of silica (SiO_2_) in the dilutions, wherein A815 and A650 represent the sample absorbances measured at wavelengths 815 nm and 650 nm, respectively:

(1) $$\left[SiO_{2}\right]\frac{mg}{L}=\;\frac{\left(A815\;-\;A650\right)\;-\;0.0278}{0.5856}$$

The concentration of silica found was used to calculate the Si content (µg) per g of dried leaf tissue (DW), accounting for the dilution factor (25), final digest volume (0.05 L), conversion factor from SiO_2_ to Si (0.467), and DW mass used (~0.0001 kg):

(2) $$\left[Si\right]\frac{µg}{g(DW)}=\frac{\left[{SiO}_{2}\right]\;\times \;25\;\times \;0.05\;\times \;0.467}{DW\;mass\;(kg)}$$

### 2.4. Determination of Plant Growth

#### 2.4.1. Leaf Chlorophyll Content Index

The relative greenness and chlorophyll concentration of leaves of the basil plants were non-destructively estimated before harvest using a chlorophyll content meter (CCM-200; Opti-Sciences, Hudson, NH, USA). A leaf chlorophyll content index (CCI) measurement was taken from a leaf on three randomly chosen plants within a replicate pot and averaged to obtain a single CCI value. The last fully expanded, unshaded leaves were selected, and measurements were taken at approximately the center of a leaf without overlapping the midrib. All replicate pots were used from each trial (8 replicates for the 0 mg/L application rate and 13 replicates for each of the other rates).

#### 2.4.2. Plant Height

The plant height of the basil plants was determined before harvest by measuring the highest point of the tallest plant in each replicate pot, from where the stem met the growing media, using a ruler. All replicate pots were used from each trial (8 replicates for the 0 mg/L application rate and 13 replicates for each of the other rates).

#### 2.4.3. Fresh Weight

The fresh weight (FW) of the basil plants was determined at harvest by cutting 5 plants from a pot where the stems met the growing media and weighing them together on a balance (PM16-N; Mettler Toledo, Mississauga, ON, Canada). Five replicate pots for the 0 mg/L application rate and 10 replicate pots for each of the other rates were used from each trial.

### 2.5. Determination of Postharvest Quality During Cold Storage

The harvested plants used to determine FW were packaged into polyethylene terephthalate clamshells (19.5 × 17.0 × 7.0 cm). Each clamshell contained a replicate, consisting of 5 plants (leaves attached to stem) from the same pot, cut at the growing media level. Five replicate pots for the 0 mg/L application rate and 10 replicate pots for each of the other rates were used from each trial. The clamshells were stored in a walk-in fridge at an average temperature of 4.0 ± 0.01 °C and RH of 76.3 ± 0.06%, as recorded by a data logger (HOBO U12-013; HOBO Data Loggers, Bourne, MA, USA) throughout the storage period.

#### 2.5.1. Fresh Weight Loss

The initial weight for each replicate was measured at harvest and then re-weighed every two days during cold storage to get the new weight for a given day using a balance (PM16-N; Mettler Toledo, Mississauga, ON, Canada). Fresh weight loss (FWL) was calculated as the percentage of weight change compared to the initial weight:

(3) $$FWL\left(\%\right)=\;\frac{\left(initial\;weight\;-\;new\;weight\right)}{initial\;weight}\;\times 100$$

#### 2.5.2. Shelf Life

**Figure 1:**
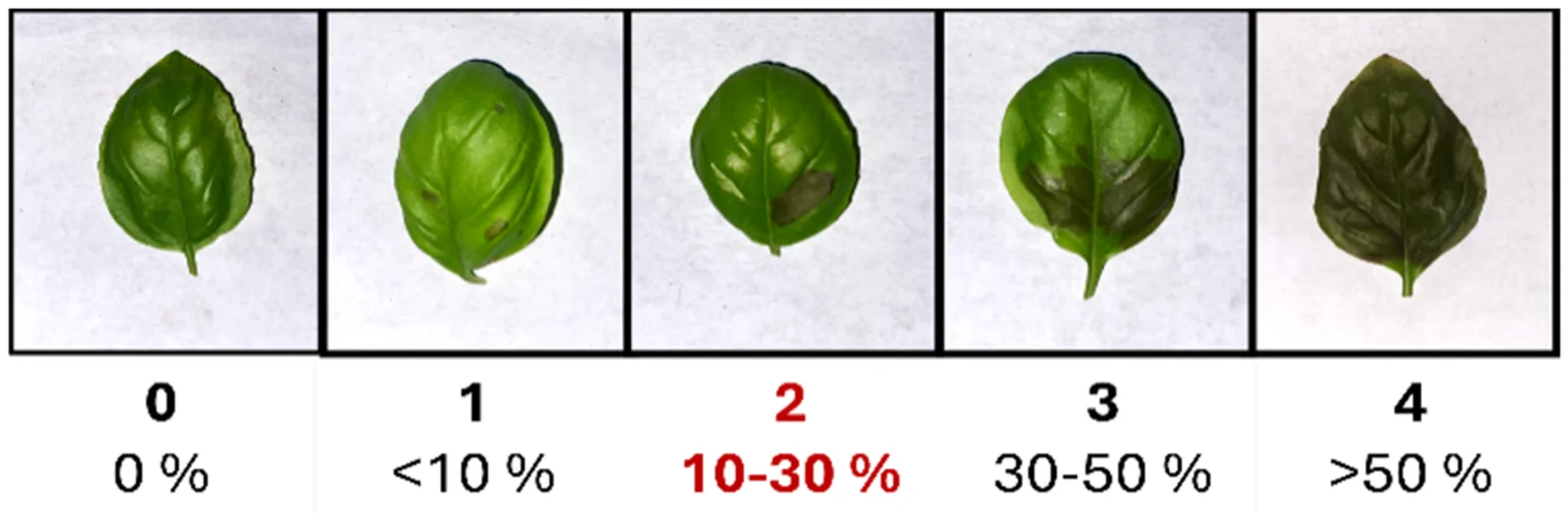
CII scoring scale with the percentage, or range of percentages, of necrotic leaf area for each score, with a visual example of each. The score of 2 in red indicates when the scores begin to be commercially unacceptable, and the replicate is past its shelf life. Note: From Ly & Zheng, 2025 [9].

Chilling injury index (CII) scores for the relative severity of chilling injury were determined for each replicate on a scale of 0–4, adapted from Wongsheree et al. [15], based on necrotic leaf area (NLA), the percentage of leaf surface covered by dark spots from a visually representative leaf (Figure 1). CII scores were determined visually by the same evaluator every two days during cold storage for the first 16 days, and then daily from day 17 onwards, until the majority (>50%) of each treatment group received an unacceptable score. A score of ≥2 was considered commercially unacceptable.

### 2.6. Statistical Analysis

All four trials were conducted using a completely randomized design (CRD). Data was analyzed using Prism ver. 11.0.2 (GraphPad Software Inc., San Diego, CA, USA).

One-way analysis of variance (ANOVA) was performed to evaluate the effect of Si application rate on leaf tissue Si content, leaf CCI, plant height, and FW. This was followed by Tukey’s test when significant effects were detected to determine significant differences (*p* < 0.05) among treatment groups within each trial. Two-way repeated-measures ANOVA, based on the general linear model, was performed to evaluate the effects of Si application rate, time (days in cold storage), and their interaction on FWL. Time was treated as the repeated-measures factor, with each replicate measured on each observation day during storage. When significant effects were detected, Tukey’s test was used to determine significant differences (*p* < 0.05) in FWL (%) among treatment groups within each storage duration for a given trial using untransformed values. Assumptions of normality and homogeneity of variance were evaluated using residual diagnostics (Q-Q, residual, and homoscedasticity plots).

Kaplan–Meier survival analysis was performed on the CII scores recorded during cold storage to determine shelf life, followed by a log-rank test with Holm–Šídák correction to determine significant differences (*p* < 0.05) in shelf life among treatments within each trial. Kaplan–Meier survival analysis estimates the probability of an event occurring over time and has been used to determine the shelf life of perishable commodities [16]. The event (shelf-life endpoint) was defined as when a sample is first observed to receive an unacceptable CII score of ≥2. Any samples that remained acceptable until the end of cold storage were censored. The time-to-event (number of days to receive an unacceptable CII score) was used to plot survival curves that estimate the probability of each treatment group remaining acceptable during postharvest cold storage. The median shelf life was estimated to be the time at which the survival probability reached 50%.

## 3. Results

### 3.1. Effect of Silicon Treatments on Leaf Tissue Silicon Content

**Figure 2:**
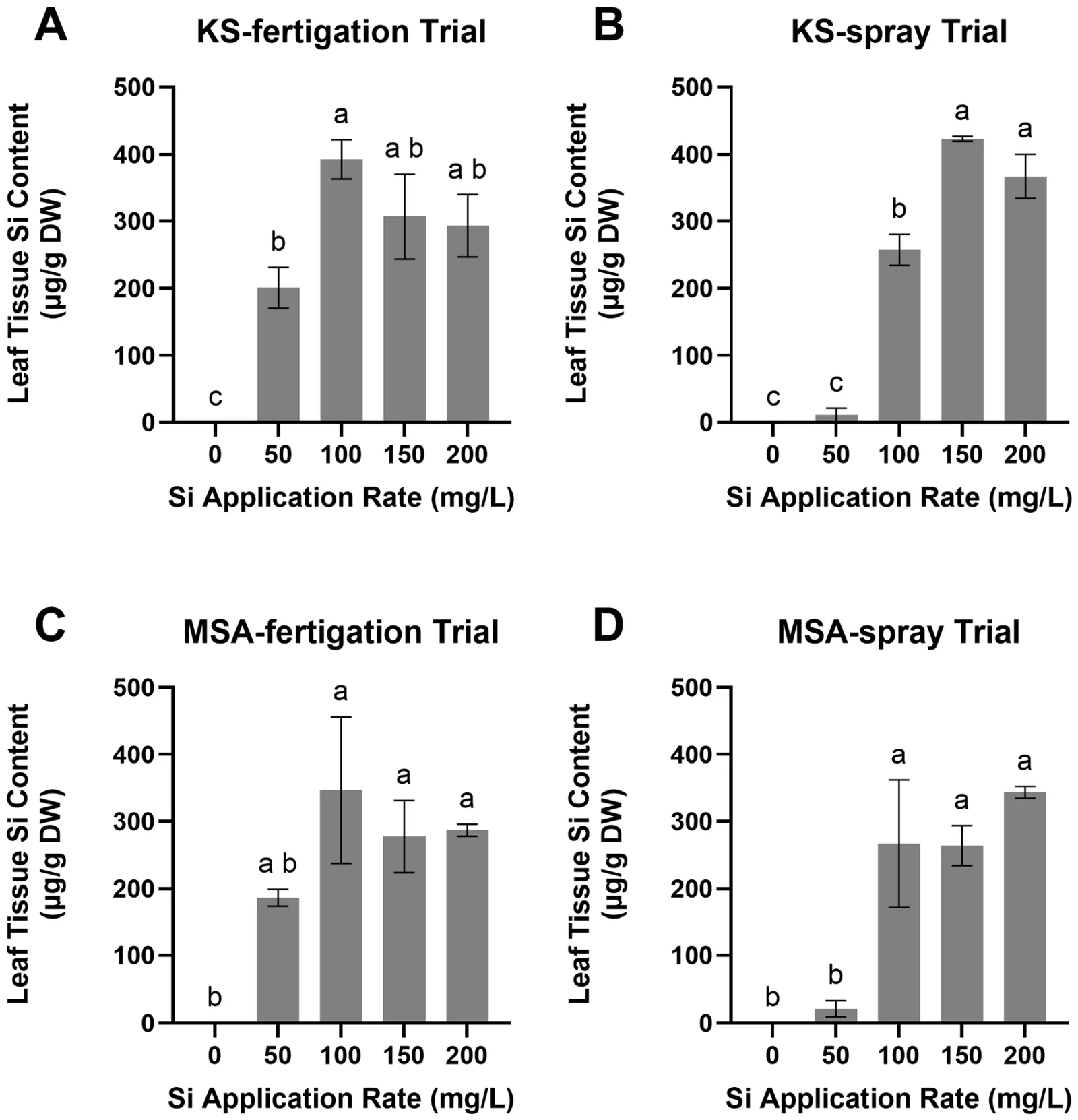
Leaf tissue silicon content per gram of dried leaf tissue (DW) of Si-treated basil at harvest in response to different application strategies. Data are means ± SEM (*n* = 3). Values from the same trial that do not share any letters are significantly different (*p* < 0.05) according to Tukey’s test. (**A**) KS-fertigation trial = rootzone irrigation every 1–2 days using nutrition solutions containing potassium silicate at different rates, (**B**) KS-spray trial = weekly foliar spray using tap water containing potassium silicate at different rates, (**C**) MSA-fertigation trial = rootzone irrigation every 1–2 days using nutrition solutions containing monosilicic acid at different rates, (**D**) MSA-spray trial = weekly foliar spray using tap water containing monosilicic acid at different rates.

Basil treated with Si had significantly greater Si content in leaf tissues compared to the untreated control groups when using application rates of at least 50 mg/L under the KS-fertigation strategy or 100 mg/L under the other strategies (Figure 2). There were no further significant increases in Si content using an application rate > 150 mg/L under the KS-spray strategy or >100 mg/L under the other strategies compared to lower rates, suggesting that rates of 100–150 mg/L are sufficient for maximizing Si uptake in basil. The lowest rate to achieve the greatest significant increase in Si content for each strategy was 100 mg/L for KS-fertigation, MSA-fertigation, and MSA-spray, and 150 mg/L for KS-fertigation. The Si content in control groups (0 mg/L) produced corrected absorbances that were below zero and could not yield a quantifiable Si concentration, demonstrating the negligible availability and uptake of Si by basil without supplementation.

### 3.2. Effect of Silicon Treatments on Plant Growth

#### 3.2.1. Leaf Chlorophyll Content Index

**Figure 3:**
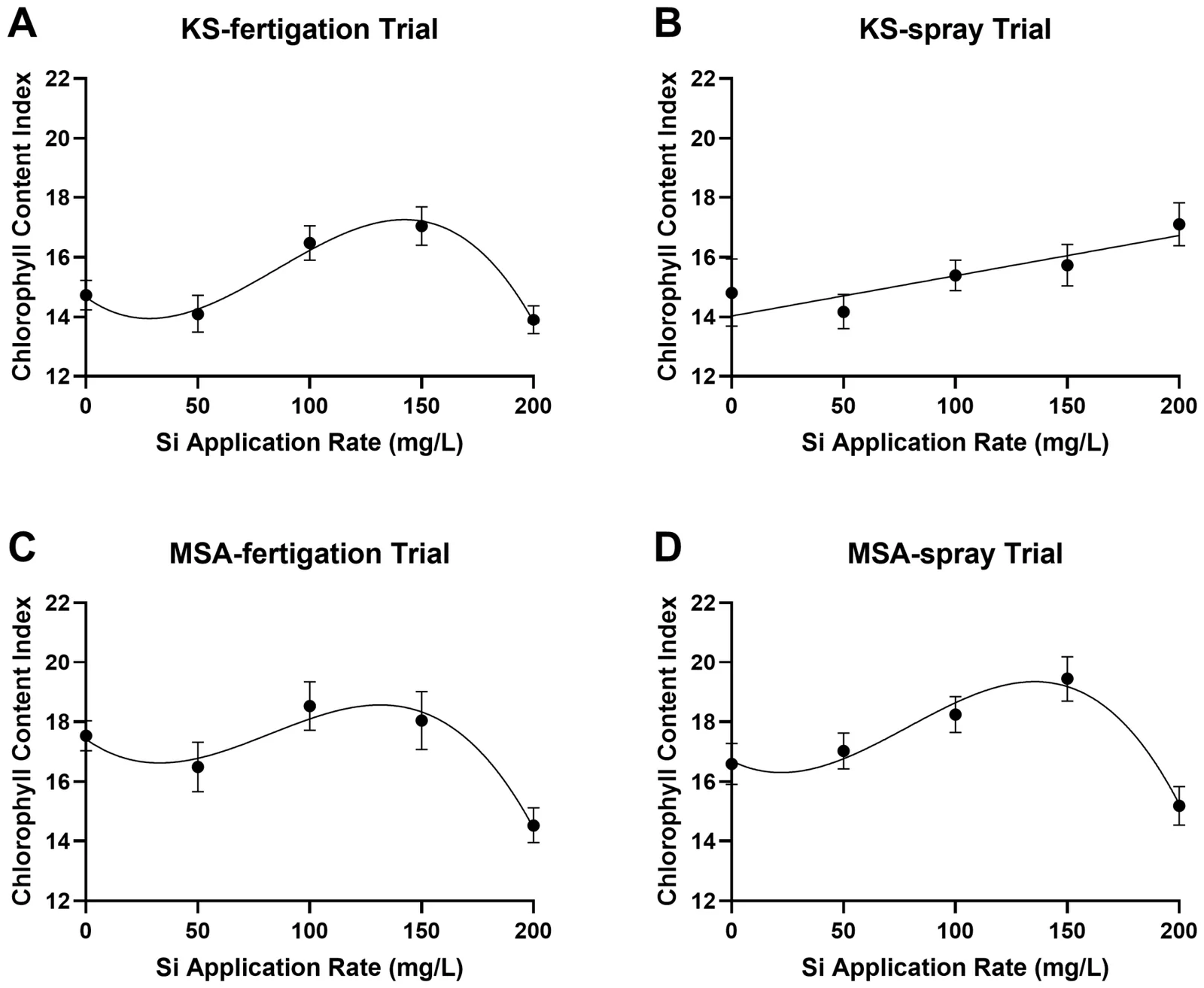
Relationship between Si application rate and leaf chlorophyll content index (CCI) at harvest under different treatment strategies. Data points are means ± SEM (*n* = 8 for 0 mg/L and *n* = 13 for all other application rates). (**A**) KS-fertigation trial = rootzone irrigation every 1–2 days using nutrition solutions containing potassium silicate at different rates, (**B**) KS-spray trial = weekly foliar spray using tap water containing potassium silicate at different rates, (**C**) MSA-fertigation trial = rootzone irrigation every 1–2 days using nutrition solutions containing monosilicic acid at different rates, (**D**) MSA-spray trial = weekly foliar spray using tap water containing monosilicic acid at different rates.

Basil leaf CCI responded to the Si application rate, either cubically (Figure 3A,C,D)—increasing in leaf CCI as the application rate increased up to 150 mg/L before dropping—or linearly increasing (Figure 3B).

#### 3.2.2. Plant Height

**Figure 4:**
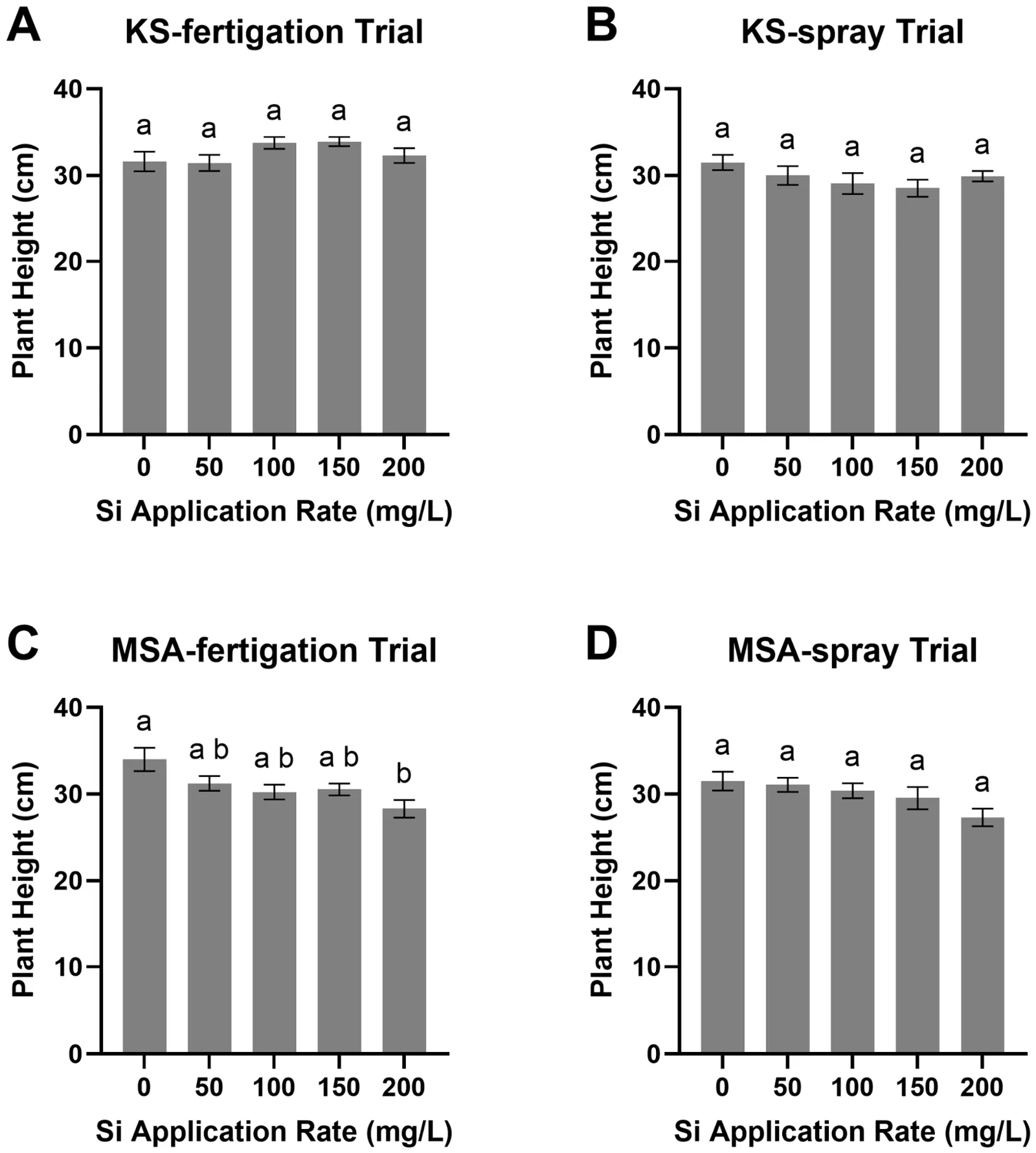
Height of Si-treated basil at harvest in response to different Si application strategies. Data are means ± SEM (*n* = 8 for 0 mg/L application rate and *n* = 13 for all other rates). Values from the same trial that do not share any letters are significantly different (*p* < 0.05) according to Tukey’s test. (**A**) KS-fertigation trial = rootzone irrigation every 1–2 days using nutrition solutions containing potassium silicate at different rates, (**B**) KS-spray trial = weekly foliar spray using tap water containing potassium silicate at different rates, (**C**) MSA-fertigation trial = rootzone irrigation every 1–2 days using nutrition solutions containing monosilicic acid at different rates, (**D**) MSA-spray trial = weekly foliar spray using tap water containing monosilicic acid at different rates.

Basil treated with Si had no significant differences in height compared to untreated controls, regardless of application rate, under every strategy except the MSA-fertigation trial (Figure 4). In the MSA-fertigation trial, there was a 17% reduction in plant height using the 200 mg/L application rate compared to the untreated controls (32 ± 1.1 cm), suggesting that higher rates could have a negative effect on plant growth.

#### 3.2.3. Fresh Weight

**Figure 5:**
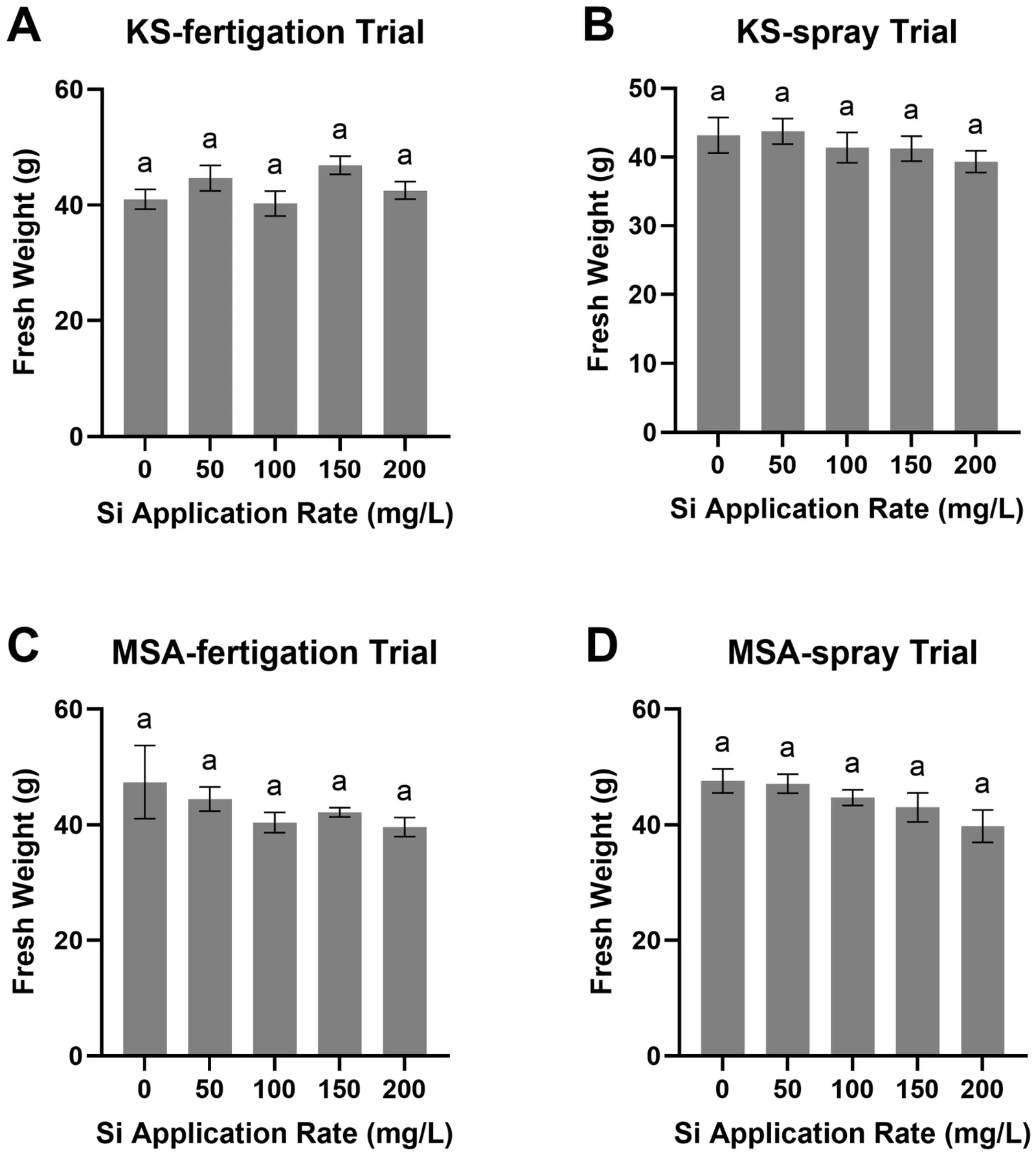
Fresh weight of Si-treated basil at harvest in response to different application strategies. Data are means ± SEM (*n* = 5 for 0 mg/L application rate and *n* = 10 for all other rates). Values from the same trial that do not share any letters are significantly different (*p* < 0.05) according to Tukey’s test. FW consists of stems cut at growing media level with leaves attached. (**A**) KS-fertigation trial = rootzone irrigation every 1–2 days using nutrition solutions containing potassium silicate at different rates, (**B**) KS-spray trial = weekly foliar spray using tap water containing potassium silicate at different rates, (**C**) MSA-fertigation trial = rootzone irrigation every 1–2 days using nutrition solutions containing monosilicic acid at different rates, (**D**) MSA-spray trial = weekly foliar spray using tap water containing monosilicic acid at different rates.

There were no significant differences in FW among treatments, regardless of application rate or strategy (Figure 5). The FW (mean ± SEM) was 43 ± 0.9 g in the KS-fertigation trial, 42 ± 0.9 g in the KS-spray trial, 42 ± 1.0 g in the MSA-fertigation trial, and 44 ± 1.0 g in the MSA-spray trial.

### 3.3. Effect of Silicon Treatments on Postharvest Quality During Cold Storage

#### 3.3.1. Fresh Weight Loss

Basil treated with Si had no significant differences in FWL compared to untreated controls, regardless of application rate, throughout most of the cold storage test except for individual days in three of the trials (Table 3, Table 4, Table 5 and Table 6). In the KS-fertigation trial, the FWL was 1.7 and 1.7% lower using the 150 and 200 mg/L application rates, respectively, compared to the untreated controls (4.8 ± 0.4%) on day 16 of cold storage (Table 3). In the KS-spray trial, the FWL was 0.8% lower using the 50 mg/L application rate compared to the untreated controls (7.2 ± 0.47%) on day 20 of cold storage (Table 4). In the MSA-spray trial, the FWL was 1.3% lower using the 50 mg/L application rate compared to the untreated controls (3.2 ± 0.18%) on day 14 of cold storage (Table 6). On the day the cold storage test ended for each trial, once the majority of all the treatment groups were no longer commercially acceptable, the FWL ranged between 5 and 7% on day 20 in the KS-fertigation trial, 6 and 10% on day 24 in the KS-spray trial, 4 and 6% on day 20 in the MSA-fertigation trial (Table 5), and 6 and 7% on day 24 in the MSA-spray trial.

#### 3.3.2. Shelf Life

Basil treated with Si had no significant differences in shelf life on the basis of necrotic leaf area compared to the untreated controls, regardless of application rate, under every strategy except the KS-fertigation trial (Table 7). In the KS-fertigation trial, the 50 and 100 mg/L application rates reduced shelf life by 2.5–3 days compared to the untreated controls (20 days).

## 4. Discussion

Studies have suggested that providing supplemental Si can be beneficial for plant growth and stress tolerance; however, results have varied across different studies on basil as well as among different crops. Our current study did not observe significant improvements in plant growth or postharvest quality during cold storage; as such, a correlation between the efficacy of Si treatments and Si content in leaf tissues was also not found.

Numerous studies have investigated the deposition of Si within plants; however, the sites and proposed molecular uptake mechanisms have yet to be fully characterized among different species. This is particularly the case for dicots like basil, which are generally non- or micro-accumulators of Si. While our study did not localize the sites of Si deposition or how it is transported, Si application rates of 100–150 mg/L were sufficient to significantly increase Si content in leaf tissues compared to untreated controls under the strategies we tested; however, the amounts did not exceed 1000 mg/kg. This further confirms the classification of basil as a non- or micro-accumulator of Si, as consistent with findings from Awan et al. [11] and Li et al. [10]. A potential explanation for the lack of improvements in growth or postharvest quality may be that a minimum threshold amount of Si is necessary to induce positive effects. Certain benefits from Si are better observed in high-accumulators and may be attributed to a greater deposition of Si in cell walls to reinforce them, such as by acting as a physical barrier against pests and fungi or by thickening the cuticle to reduce water loss and wilting from transpiration [17,18]. Basil may not accumulate sufficient Si to observe such benefits. The observed growth and stress tolerance benefits for non- or micro-accumulators are speculated to be from Si-mediated signalling or Si-facilitated mechanisms which increase antioxidant potential, photosynthetic capacity, and uptake of other macro- and micronutrients [17]. However, these activities may be limited by Si bioavailability or require a minimum Si concentration to initiate. Further studies investigating Si transport mechanisms and eventual sites of deposition could improve our understanding of how Si imparts benefits.

Si is suggested to increase or preserve chlorophyll concentrations, although there is no clear evidence of Si entering organelles linked to physiological processes like photosynthesis [17,19,20]. While Si treatments are commonly found to positively influence chlorophyll content in crops under abiotic or biotic stress conditions, this effect is not as pronounced or as consistently observed in the absence of stress [20,21,22]. Our study found that basil leaf CCI increased as the application rate increased up to 150 mg/L, after which it dropped, except for the KS-spray, which increased linearly as the Si application rate increased. These results are consistent with those of Tebow et al. [13] and Awan et al. [11], who both saw a significant increase in basil chlorophyll content using Si applications. However, a study on maize saw no effect on chlorophyll content, regardless of whether the plants were under chilling stress or not [23]. We also found no effect on FW or plant height, apart from a reduction in plant height when using the 200 mg/L application rate in the KS-fertigation trial. Si is largely considered not to be phytotoxic, but there may be potential for adverse effects at certain concentrations or for certain crops. Tebow et al. [13] also found that Si applications had no effect on the FW or plant height of potted basil, as well as no effect on the plant heights of other herbs including parsley, rosemary, and thyme. However, morphology was distorted by Si treatments in parsley. Contrary to these findings, studies by Awan et al. [11] and Li et al. [10] both observed increased plant heights and FW in Si-treated basil. These results show the inconsistent effects Si has during plant growth and development. Effects from Si treatments may only be observed or more apparent after exposure to stress. Further studies should be done to better understand the mechanisms involved to improve the impact and consistency of these potential benefits, as well as investigating the effects of Si treatments on basil after exposure to specific, isolated stressors during cultivation rather than just postharvest chilling stress.

It is posited that CI symptoms are, at least in part, the result of oxidative damage from the accumulation of reactive oxygen species (ROS) during chilling stress. Disruption of redox homeostasis from chilling stress can result in an imbalance between ROS production and the scavenging capacity of antioxidants, promoting lipid peroxidation and damage to cellular membranes and other components, thus accelerating tissue senescence and water loss [8]. This could give rise to common CI symptoms like leaf necrosis and wilting from FWL, key visual indicators for commercial quality. Research has shown that Si applications can increase the chilling tolerance of many cold-sensitive crops to alleviate CI symptoms during cultivation or postharvest cold storage. The mechanisms by which Si confers chilling resistance and reduces CI symptoms are still being investigated; however, several studies on different Si-treated crops under chilling stress found that they enhance antioxidant capacity to limit ROS accumulation [24,25,26,27]. This was observed with the increased activity of antioxidants, including peroxidase, superoxide dismutase, and catalase, while ROS and end-products of lipid peroxidation, such as hydrogen peroxide and malondialdehyde, were reduced. Studies conducted postharvest have also shown similar effects, although these studies are mainly limited to fruits [5,6]. Despite evidence that basil could also benefit from preharvest Si treatments during postharvest cold storage from our previous study, Ly & Zheng [9], and suggested by findings from Li et al. [10], the current study largely did not observe significant differences in postharvest quality and shelf life compared to untreated controls; however, all groups lasted longer than expectations from the literature and long enough to increase distribution potential when transporting. The shelf life for the untreated controls lasted 18–24 days, surpassing expected estimates of around 3 days given the 4 °C storage temperature.

A conclusive explanation for the extended shelf life of the controls could not be determined by this study. Some differences between our previous study, which observed beneficial effects from Si preharvest applications, and this current one, which did not, include how the first study was conducted using organic growing practices and smaller pot sizes [9]. These conditions led to much smaller plants at harvest compared to plants from this current study, which were provided a nutrient solution and more room to grow. Potential differences in nutritional status and structural development could lead to the controls in this study lasting longer during cold storage. Another potential reason for the slow development of CI symptoms and extended shelf life during the cold storage test could be drought stress experienced during cultivation. On hot, sunny days during cultivation, the potted basil would occasionally experience slight wilting and require additional watering to recover. These events could have been sufficient to induce protective mechanisms in the untreated basil against oxidative damage during chilling stress by increasing antioxidant enzymes, which reduce ROS accumulation, as observed in drought studies on cucumber [28], maize [29], and tomato seedlings [30]. More experimentation to exclude potential contributing factors may be necessary to confirm the efficacy of Si applications for postharvest cold stress tolerance in basil. Future studies may also investigate drought-hardening as another potential method.

## 5. Conclusions

No significantly positive effects from Si treatments were observed at harvest. Excluding minor significant reductions in FWL on singular days during three of the trials, and a slightly shorter shelf life for some treatment groups in the KS-fertigation trial, Si treatments had little effect on postharvest quality during cold storage compared to the untreated controls. The shelf life for all the treatment groups ranged from 17–24 days, surpassing the estimates of 1–3 days in the literature for basil stored at similar temperatures and would allow for greater distribution. However, with the controls lasting 18–24 days, there was likely an influencing factor beyond the scope of this study impacting the untreated basil that we cannot determine conclusively, such as drought stress. Further investigations are necessary to isolate and confirm the efficacy of Si alone.

From our observations during this study, growers may need to be cautious about using Si application rates >150 mg/L, as it could potentially have negative effects during cultivation, as seen for plant height in the MSA-fertigation trial. Furthermore, Si uptake did not significantly increase beyond this rate. The lowest rate necessary to provide the greatest significant increase in leaf Si content for each strategy was 100 mg/L for KS-fertigation, MSA-fertigation, and MSA-spray or 150 mg/L for KS-spray. Observed effectiveness of Si treatments has varied among different studies; before adopting any Si application strategies commercially on a large scale, basil growers should run trials on their own crops to evaluate the potential benefits.

## Figures and Tables

**Table 1:** Silicon application strategy used in each trial.

Trial	Strategy Name	Treatment Solution	Application Method
1	KS-fertigation	Nutrient solution containing KS	Rootzone fertigation
2	KS-spray	Tap water containing KS	Weekly foliar spray
3	MSA-fertigation	Nutrient solution containing MSA	Rootzone fertigation
4	MSA-spray	Tap water containing MSA	Weekly foliar spray

KS = potassium silicate, MSA = monosilicic acid.

**Table 2:** Amount of potassium silicate (KS) and monosilicic acid (MSA) used per litre of nutrient solution or tap water for each application rate.

Si Application Rate (mg/L)	KS Used (mL/L)	MSA Used (mL/L)
0	0.00	0.00
50	1.43	0.52
100	2.86	1.04
150	4.29	1.56
200	5.72	2.08

KS = potassium silicate, MSA = monosilicic acid.

**Table 3:** Fresh weight loss (FWL) of basil plants (%) during the cold storage test at 4 °C in the KS-fertigation strategy trial.

Days	Si Application Rate (mg/L)				
	0	50	100	150	200
02	0.0 ± 0.0 a	0.0 ± 0.0 a	0.0 ± 0.0 a	0.0 ± 0.0 a	0.1 ± 0.0 a
04	0.8 ± 0.4 a	0.7 ± 0.2 a	1.0 ± 0.2 a	0.3 ± 0.1 a	0.4 ± 0.2 a
06	1.4 ± 0.4 a	0.8 ± 0.2 a	1.2 ± 0.2 a	0.7 ± 0.2 a	0.7 ± 0.2 a
08	1.8 ± 0.4 a	1.2 ± 0.3 a	1.7 ± 0.2 a	0.9 ± 0.2 a	1.1 ± 0.3 a
10	2.1 ± 0.5 a	1.4 ± 0.3 a	2.0 ± 0.3 a	1.1 ± 0.2 a	1.4 ± 0.3 a
12	2.5 ± 0.4 a	1.7 ± 0.3 a	2.3 ± 0.3 a	1.4 ± 0.2 a	1.7 ± 0.3 a
14	2.7 ± 0.4 ab	2.2 ± 0.3 ab	3.3 ± 0.4 b	1.7 ± 0.2 a	2.3 ± 0.3 ab
16	4.8 ± 0.4 b	4.7 ± 0.4 b	4.5 ± 0.5 ab	3.1 ± 0.3 a	3.2 ± 0.3 a
18	6.6 ± 0.6 a	6.1 ± 0.5 a	5.6 ± 0.5 a	5.2 ± 0.4 a	4.6 ± 0.3 a
20	6.9 ± 0.6 a	6.6 ± 0.4 a	6.2 ± 0.6 a	5.6 ± 0.4 a	5.0 ± 0.3 a

Data are means ± SEM (*n* = 5 for 0 mg/L application rate and *n* = 10 for all other rates). Values from the same day that do not share any letters are significantly different (*p* < 0.05) according to Tukey’s test. KS-fertigation trial = rootzone irrigation every 1–2 days using nutrition solutions containing potassium silicate at different rates.

**Table 4:** Fresh weight loss (FWL) of basil plants (%) during the cold storage test at 4 °C in the KS-spray strategy trial.

Days	Si Application Rate (mg/L)				
	0	50	100	150	200
02	0.0 ± 0.0 a	0.0 ± 0.0 a	0.0 ± 0.0 a	0.0 ± 0.0 a	0.0 ± 0.0 a
04	0.7 ± 0.4 a	0.3 ± 0.1 a	0.5 ± 0.2 a	0.6 ± 0.3 a	0.4 ± 0.2 a
06	1.3 ± 0.6 a	0.8 ± 0.3 a	0.8 ± 0.3 a	1.4 ± 0.4 a	1.0 ± 0.4 a
08	1.8 ± 0.6 a	1.2 ± 0.3 a	1.7 ± 0.7 a	2.8 ± 0.6 a	1.6 ± 0.6 a
10	2.2 ± 0.8 a	1.5 ± 0.4 a	2.3 ± 0.8 a	3.6 ± 0.8 a	2.2 ± 0.7 a
12	2.8 ± 0.7 a	1.9 ± 0.4 a	2.9 ± 0.8 a	4.3 ± 0.8 a	2.6 ± 0.7 a
14	3.2 ± 0.5 a	2.4 ± 0.4 a	3.6 ± 0.9 a	4.9 ± 0.9 a	2.5 ± 1.0 a
16	3.7 ± 0.5 a	2.9 ± 0.4 a	4.0 ± 0.9 a	5.9 ± 1.0 a	4.1 ± 0.8 a
18	4.5 ± 0.5 a	4.3 ± 0.6 a	5.8 ± 1.0 a	7.3 ± 1.1 a	5.4 ± 0.9 a
20	5.2 ± 0.4 a	4.7 ± 0.5 a	6.3 ± 1.0 a	7.9 ± 1.0 a	5.8 ± 0.9 a
23 *	6.3 ± 0.3 a	5.4 ± 0.5 a	7.3 ± 0.9 a	8.6 ± 0.9 a	6.9 ± 0.8 a
24	7.2 ± 0.5 b	6.4 ± 0.6 a	8.0 ± 0.8 ab	9.6 ± 0.9 ab	7.7 ± 0.8 ab

Data are means ± SEM (*n* = 5 for 0 mg/L application rate and *n* = 10 for all other rates). Values from the same day that do not share any letters are significantly different (*p* < 0.05) according to Tukey’s test. * Due to access to storage facility being restricted on day 22, measurements were taken on day 23 instead. KS-spray trial = weekly foliar spray using tap water containing potassium silicate at different rates.

**Table 5:** Fresh weight loss (FWL) of basil plants (%) during the cold storage test at 4 °C in the MSA-fertigation strategy trial.

Days	Si Application Rate (mg/L)				
	0	50	100	150	200
02	0 ± 0.0 a	0.2 ± 0.2 a	0.0 ± 0.0 a	0.0 ± 0.0 a	0.0 ± 0.0 a
04	0.2 ± 0.2 a	0.7 ± 0.1 a	0.7 ± 0.2 a	0.4 ± 0.1 a	0.6 ± 0.1 a
06	0.8 ± 0.3 a	0.9 ± 0.2 a	1.1 ± 0.3 a	0.8 ± 0.1 a	0.9 ± 0.1 a
08	1.0 ± 0.2 a	1.1 ± 0.2 a	1.3 ± 0.3 a	1.0 ± 0.2 a	1.1 ± 0.1 a
10	1.3 ± 0.1 a	1.2 ± 0.2 a	2.1 ± 0.6 a	1.1 ± 0.2 a	1.3 ± 0.1 a
12	1.8 ± 0.3 a	1.4 ± 0.2 a	2.0 ± 0.3 a	1.4 ± 0.2 a	1.5 ± 0.1 a
14	2.5 ± 0.4 a	2.1 ± 0.2 a	2.4 ± 0.2 a	1.9 ± 0.2 a	2.1 ± 0.2 a
16	4.0 ± 0.6 a	2.5 ± 0.2 a	3.3 ± 0.2 a	2.7 ± 0.3 a	2.6 ± 0.2 a
18	5.9 ± 0.8 a	3.6 ± 0.3 a	4.7 ± 0.3 a	3.7 ± 0.3 a	3.8 ± 0.3 a
20	6.0 ± 0.7 ab	3.8 ± 0.3 a	5.1 ± 0.3 b	4.1 ± 0.3 ab	4.2 ± 0.3 ab

Data are means ± SEM (*n* = 5 for 0 mg/L application rate and *n* = 10 for all other rates). Values from the same day that do not share any letters are significantly different (*p* < 0.05) according to Tukey’s test. MSA-fertigation trial = rootzone irrigation every 1–2 days using nutrition solutions containing monosilicic acid at different rates.

**Table 6:** Fresh weight loss (FWL) of basil plants (%) during the cold storage test at 4 °C in the MSA-spray strategy trial.

Days	Si Application Rate (mg/L)				
	0	50	100	150	200
02	0.0 ± 0.0 a	0.0 ± 0.0 a	0.0 ± 0.0 a	0.0 ± 0.0 a	0.0 ± 0.0 a
04	0.5 ± 0.5 a	0.1 ± 0.0 a	0.5 ± 0.3 a	0.8 ± 0.3 a	0.3 ± 0.2 a
06	1.1 ± 0.5 a	0.4 ± 0.2 a	0.8 ± 0.4 a	1.1 ± 0.4 a	0.6 ± 0.3 a
08	1.6 ± 0.5 a	1.0 ± 0.2 a	1.2 ± 0.4 a	1.7 ± 0.3 a	1.2 ± 0.3 a
10	2.3 ± 0.5 a	1.3 ± 0.3 a	1.6 ± 0.4 a	2.0 ± 0.4 a	1.4 ± 0.3 a
12	2.7 ± 0.3 a	1.6 ± 0.2 a	1.9 ± 0.4 a	2.5 ± 0.4 a	1.8 ± 0.3 a
14	3.2 ± 0.2 b	1.9 ± 0.4 a	2.0 ± 0.4 ab	3.0 ± 0.4 ab	2.1 ± 0.3 ab
16	3.5 ± 0.1 a	2.4 ± 0.3 a	2.5 ± 0.4 a	3.7 ± 0.5 a	2.9 ± 0.4 a
18	4.6 ± 0.6 a	3.5 ± 0.3 a	3.3 ± 0.5 a	5.0 ± 0.4 a	4.1 ± 0.5 a
20	4.9 ± 0.6 ab	3.7 ± 0.2 a	3.8 ± 0.5 ab	5.4 ± 0.5 b	4.6 ± 0.5 ab
23 *	6.3 ± 0.6 a	4.7 ± 0.3 a	4.6 ± 0.6 a	6.3 ± 0.5 a	5.7 ± 0.5 a
24	7.1 ± 0.7 a	6.5 ± 0.3 a	5.7 ± 0.7 a	7.5 ± 0.5 a	6.7 ± 0.5 a

Data are means ± SEM (*n* = 5 for 0 mg/L application rate and *n* = 10 for all other rates). Values from the same day that do not share any letters are significantly different (*p* < 0.05) according to Tukey’s test. * Due to access to storage facility being restricted on day 22, measurements were taken on day 23 instead. MSA-spray trial = weekly foliar spray using tap water containing monosilicic acid at different rates.

**Table 7:** Median shelf life of Si-treated basil during postharvest cold storage at 4 °C under different application strategies.

Trial Strategy	Si Application Rate (mg/L)	Median Shelf Life (Days)
KS-fertigation	0	20 a
	50	17 b
	100	17.5 b
	150	18 ab
	200	18 ab
KS-spray	0	24 a
	50	24 a
	100	21 a
	150	23 a
	200	24 a
MSA-fertigation	0	18 a
	50	19.5 a
	100	19 a
	150	19 a
	200	20 a
MSA-spray	0	24 a
	50	22 a
	100	22 a
	150	23.5 a
	200	24 a

Data are Kaplan–Meier estimates of survival (length of shelf life), based on acceptable CII scores, by treatment group (*n* = 5 for 0 mg/L application rate and *n* = 10 for all other rates). Values for median shelf life that do not share any letters within a trial are significantly different (*p* < 0.05) according to log-rank test with Holm–Šídák correction. KS-fertigation trial = rootzone irrigation every 1–2 days using nutrition solutions containing potassium silicate at different rates, KS-spray trial = weekly foliar spray using tap water containing potassium silicate at different rates, MSA-fertigation trial = rootzone irrigation every 1–2 days using nutrition solutions containing monosilicic acid at different rates, MSA-spray trial = weekly foliar spray using tap water containing monosilicic acid at different rates.

## Data Availability

The raw data supporting the conclusions of this article will be made available by the authors upon request.

## Abbreviations

The following abbreviations are used in this manuscript:

CCI	Chlorophyll content index
CI	Chilling injury
CII	Chilling injury index
DI	Deionized
DLI	Daily light integral
DW	Dried leaf tissue
FW	Fresh weight
FWL	Fresh weight loss
KS	Potassium silicate
MSA	Monosilicic acid
NLA	Necrotic leaf area
RH	Relative humidity
ROS	Reactive oxygen species
Si	Silicon
SiO_2_	Silica
